# Relationship between serum prostate-specific antigen and age in cadavers

**DOI:** 10.1177/2050312120958212

**Published:** 2020-09-16

**Authors:** Hajime Tsuboi, Daisuke Miyamori, Noboru Ishikawa, Hiroaki Ichioka, Hiroshi Ikegaya

**Affiliations:** 1 Department of Forensic Medicine, Graduate School of Medical Science, Kyoto Prefectural University of Medicine, Kyoto, Japan; 2 Department of General Medicine, Graduate School of Medical Science, Hiroshima University, Hiroshima, Japan; 3 Department of Histology and Developmental Biology, Tokyo Dental College, Chiyoda-ku, Japan

**Keywords:** Prostate-specific antigen, age estimation, unidentified cadavers

## Abstract

**Objectives:**

An increase in number of unidentified cadavers is a growing problem. To identify these cadavers, a simple objective method is required to estimate cadaveric age. We examined the correlations between postmortem serum prostate-specific antigen levels and cadaveric age to determine whether serum prostate-specific antigen levels can be used in age estimation of unidentified cadavers.

**Methods:**

Total serum prostate-specific antigen was measured in 140 male autopsy cases aged from 0 to 94 years.

**Results:**

The serum prostate-specific antigen levels of cadavers correlated with age at death to the same degree as with the age of living individuals (r = 0.393, *P* < 0.01). Prostate-specific antigen levels also correlated with prostate weight, but not with psoas muscle index and body mass index. Cause of death did not influence postmortem serum prostate-specific antigen levels.

**Conclusion:**

Age estimation based on prostate-specific antigen provides a simple, objective, and rapid method to determine age at death estimation of cadavers, and is expected to greatly contribute to the identification of cadavers.

## Introduction

In recent years, there has been an increase in number of unidentified cadavers worldwide, and this issue is an important challenge in the field of forensic medicine.^1^ Similarly, in Japan, an increase in the incidence of unidentified cadavers has been reported.^2^ Japan is currently experiencing an aging society, with the population of elderly people (>65 years old) comprising 25% of the total population.^3^ The current proportion of households in which the elderly live alone has reached 10%, and this number increases yearly.^4^ In addition, the number of so-called “solitary deaths” is increasing.^4^ Unattended single person deaths may result in the cadaver being undiscovered for an extended period, termed “solitary death.” The great earthquake of East Japan in 2011 also resulted in many unidentified cadavers, and the identification of cadavers required a lot of time in many cases.^5,6^ An increase in cases of solitary death could lead to an increase in unidentified cadavers. In addition, further catastrophic natural disasters or indiscriminate terrorist attacks could result in many deaths, including a great number of unidentified cadavers. Under these circumstances, a method to concurrently and rapidly identify cadavers would be valuable.

Within the field of forensic medicine, cadavers have been identified utilizing DNA or dental records. However, these methods are insufficient in some cases. For example, in cases where the individual has no dental history, the head of the cadaver is damaged, there is no family or relatives from whom to derive DNA samples for comparison with the cadaver, and no personal effects with which the victim can be identified are found, identification of cadavers is extremely difficult. As a component used within the identification of cadavers, age estimation plays a significant role. If the approximate age at death of a cadaver can be estimated, the possible candidates can be narrowed down from among a list of missing persons. Currently, age estimation of cadavers is conducted using measurements of bones including the cranial bone or long bones,^7–10^ or examination of the teeth.^11,12^ However, these methods are relatively subjective, and require extensive experience and knowledge. The age estimation method that can be easily, rapidly, objectively, and inexpensively performed is not widely known in the field of forensic science.

In the present study, we focused on prostate-specific antigen (PSA). PSA is protein of molecular weight 34 kD consisting of 237 amino acids and is a serine protease belonging to the human kallikrein gene family.^13^ PSA is specifically produced in ductal epithelium cells of the prostate gland, and plays a role in promoting sperm motility when secreted in sperm. The blood concentration of PSA increases in patients with prostate cancer. The reason for this is thought to be that the amount of PSA deviated to the blood increases because the structure of ductal epithelium cells of the prostate is destroyed by prostate cancer. Therefore, PSA is widely used as a clinical tumor marker of prostate cancer.^14^ On the other hand, in the field of forensic medicine, it is known that the detection of PSA from semen gathered from a victim of sexual assault proves the presence of sperm. This method is based on the fact that PSA is secreted in sperm.^15^ It has been shown that PSA is released into the blood deviating from the prostate very slightly in healthy males, and serum PSA levels of living men correlate with the age.^16–19^ Therefore, we hypothesized that the age at death of unidentified cadavers can be estimated by measuring serum PSA levels. To date, there is no report that investigated the correlation between age at death and the serum PSA levels of cadavers.

It is thought that the serum PSA levels might be affected by specific cadaver variables such as postmortem change or cause of death. Therefore, we measured serum PSA levels of cadavers of victims which had experienced various causes of death, as well as levels within cadavers of variable postmortem intervals, and examined the correlation between serum PSA levels and age. We thereby investigated whether serum PSA levels can be used in age at death estimation of unidentified male cadavers.

## Materials and methods

### Materials

The present study is an observational retrospective study using the case data of from August 2008 to February 2015 at the Department of Forensic Medicine, Kyoto Prefectural University of Medicine, including a total of 178 male cadavers. An autopsy was performed on each cadaver, during which serum PSA levels were measured. Eight cases in which the age at death of the cadaver was unknown or where the serum PSA levels could not be measured because of a lack of specimen were excluded from the study. And 30 cases with prostate disease and insert of urinary catheter at the death were excluded. Therefore, we performed a statistical evaluation of the correlations between serum PSA levels and age, psoas muscle index (PMI), body mass index (BMI),^20^ prostate weight, and causes of death in 140 cadavers. The 140 cadavers represented deaths of people aged between 0 and 94 years old (mean ± SD: 50.6 ± 22.2 years; median: 54.5 years). The postmortem intervals of the cadavers (PMI; interval from the estimated time of death to the time of autopsy) ranged from 10 h to 3 days, and the BMI scores ranged from 11 to 39.3 (mean ± SD: 21.6 ± 4.56; median: 21.4). The causes of death ranged widely (26 cases of traumatic organ injury, 27 endogenic disease, 38 asphyxiation including hanging or drowning, 22 death by exsanguination, 11 by hypothermia, 8 by poisoning, 12 burn victims, and 26 by unknown causes). Prostatic disease was found in 17 cases (5 cases of prostate cancer, 9 benign prostatic hyperplasia, 1 acute prostatitis, and 2 chronic prostatitis) by medical record or histologic inspection. In addition, prostate weight was measured at autopsy or calculated from its size in 78 cases.

### Measurement of PSA levels

The serum samples were obtained by centrifuging the heart blood collected in the autopsy. And the serum samples were stored at −80°C until use. We measured serum total PSA levels of cadavers using the chemiluminescence immunoassay (CLIA) method (ARCHITECT, Abbott Company, Japan).

### Correlation between postmortem serum PSA levels and age

We examined correlations between postmortem serum PSA levels and age in all 140 cadavers.

### Correlation between postmortem serum PSA levels and PMIs

We examined the influence of PMI on serum PSA levels. At first, we classified the 140 cadavers into three groups by their PMI, regardless of age at death, and examined correlations between serum PSA levels and PMI in each group. The three groups classified by PMI were (1) PMI < 24 h; (2) 24 h ⩽ PMI ⩽ 48 h; and (3) 48 h < PMI < 72 h, as previously described.^21^ Second, we classified the 140 cadavers into nine groups by age at death according to decades: (1) 0–9 years; (2) 10–19 years; (3) 20–29 years; (4) 30–39 years; (5) 40–49 years; (6) 50–59 years; (7) 60–69 years; (8) 70–79 years; and (9) >80 years. In each of these nine groups, we further classified each group into three subgroups by PMI as mentioned previously. Groups 1 (0–9 years) and 2 (10–19 years) were classified into two subgroups based on PMI, because there were no cases of over 48 h.

### Correlation between postmortem serum PSA levels and BMI

We examined the influence of BMI on postmortem serum PSA level. We examined correlations between postmortem serum PSA levels and BMI in 140 cadavers.

### Correlation between prostate weight and postmortem serum PSA levels or age

We examined correlations between prostate weight and postmortem serum PSA levels or age in the 72 cases (6 cases which had prostatic disease were excluded from previous 78 cases) among the whole 140 cases. We used the formula of an oval (median diameter × lateral diameter × top and bottom diameter × π/6) for the cases in which prostate weight was not measured at autopsy to obtain the prostate weight (g), as previously described.^22^

### Correlation between postmortem serum PSA levels and cause of death

We classified 140 cadavers (30 cases which had prostatic disease were excluded) into eight groups by cause of death: (1) traumatic organ injury; (2) endogenic disease; (3) asphyxiation including hanging or drowning; (4) exsanguination; (5) death by hypothermia; (6) poisoning; (7) burns; and 8) unknown.

### Statistical analysis

The relationship between postmortem serum PSA level and age was assessed using Spearman’s rank correlation coefficient. Root mean square error (RMSE) was calculated by the following formula

$$\mathrm{RMSE}=\sqrt{\frac{1}{n}\sum_{i=1}^{n}{\left(yi-\hat{y}i\right)}^{2}}$$

We used the Steel–Dwass method to perform multiple comparisons in the various postmortem time periods, and applied the Kruskal–Wallis method to evaluate statistical error. The relationship between postmortem serum PSA levels and BMI was assessed using Spearman’s rank correlation coefficient. The relationship between prostate weight and postmortem serum PSA levels or age was assessed using Spearman’s rank correlation coefficient. We used the Steel-Dwass method to perform multiple comparisons of serum PSA levels in the various causes of death and applied the Kruskal–Wallis method to evaluate statistical error. We used Microsoft Excel 2013 (Microsoft Corporation) and the statistical software EZR (Easy R) (R version 3.2.2),^23^ and set the level of significance in the statistical test at *P* ⩽ 0.05.

## Results

### Correlation between postmortem serum PSA levels and age

**Figure 1.**
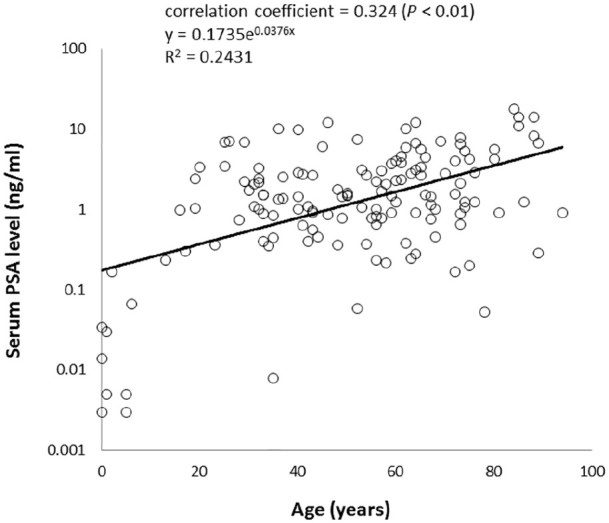
Correlation between serum prostate-specific antigen (PSA) level and age (n = 140). A significant weak positive correlation was observed (Spearman’s rank correlation coefficient). The regression curve was y = 0.1735e^0.0324x^ (R^2^ = 0.2431).

Table 1 shows the percentile values of the serum PSA levels of the 140 cadavers classified into the age groups. In general, each percentile value increased as age increased. However, this trend was not evident in the median values. The median values contained two peaks in the twenties and sixties, and reduced slightly in seventies, after which it increased again in the eighties. In addition, the distributions of serum PSA levels varied in the elderly. In these 140 cases, a significant positive correlation was observed between serum PSA levels and age (*P* < 0.01, correlation coefficient = 0.324, regression curve: y = 0.1735e^0.0376x^, x: age, y: serum PSA levels, R^2^ = 0.2431, RMSE = 1.4827) (Figure 1).

**Table 1.** Percentile values of serum PSA levels in 140 cadavers classified into age groups by decade.

Age (years)	Number	Percentile value of serum PSA level (ng/ml)			
		25%	50%	75%	95%
0–19	14	0.30	0.98	1.05	2.15
20–29	8	2.25	3.45	7.02	44.1
30–39	19	0.89	1.46	2.24	11.7
40–49	20	0.78	1.1	2.8	12.4
50–59	23	0.8	1.51	2.96	18.8
60–69	27	1.24	3.11	6.77	24.3
70–79	17	1.16	2.95	16.5	43.1
>80	12	3.50	10.3	19.2	171

PSA: prostate-specific antigen.

### Correlation between postmortem serum PSA levels and PMIs

No significant difference was observed between PMI and serum PSA levels in the 140 cadavers, regardless of age at death. Similarly, no significant difference was observed between PMI and serum PSA levels among the classified age groups.

### Correlation between postmortem serum PSA levels and BMI of cadavers

**Figure 2.**
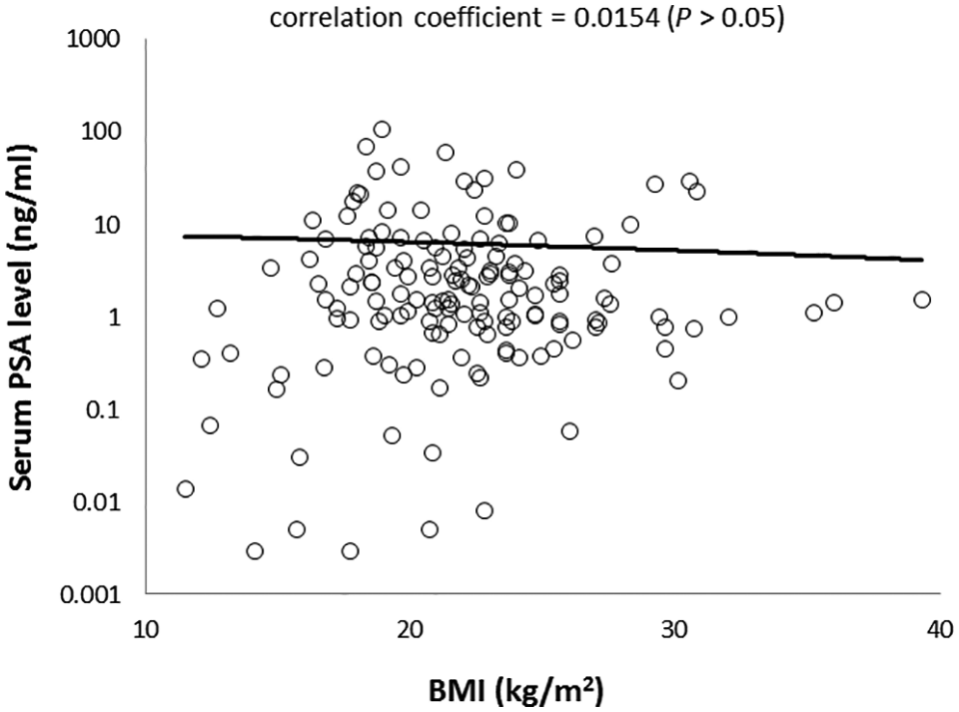
Correlation between serum prostate-specific antigen (PSA) level and body mass index (BMI) (n = 140). No significant positive correlation was observed (Spearman’s rank correlation coefficient.

No significant difference was observed between BMI and postmortem serum PSA levels in the 153 cadavers, regardless of age (*P* = 0.78, correlation coefficient = −0.0154) (Figure 2). Furthermore, no significant difference was observed between BMI and *postmortem* serum PSA levels among the age classified groups.

### Correlation between prostate weight and postmortem serum PSA levels or age

**Figure 3.**
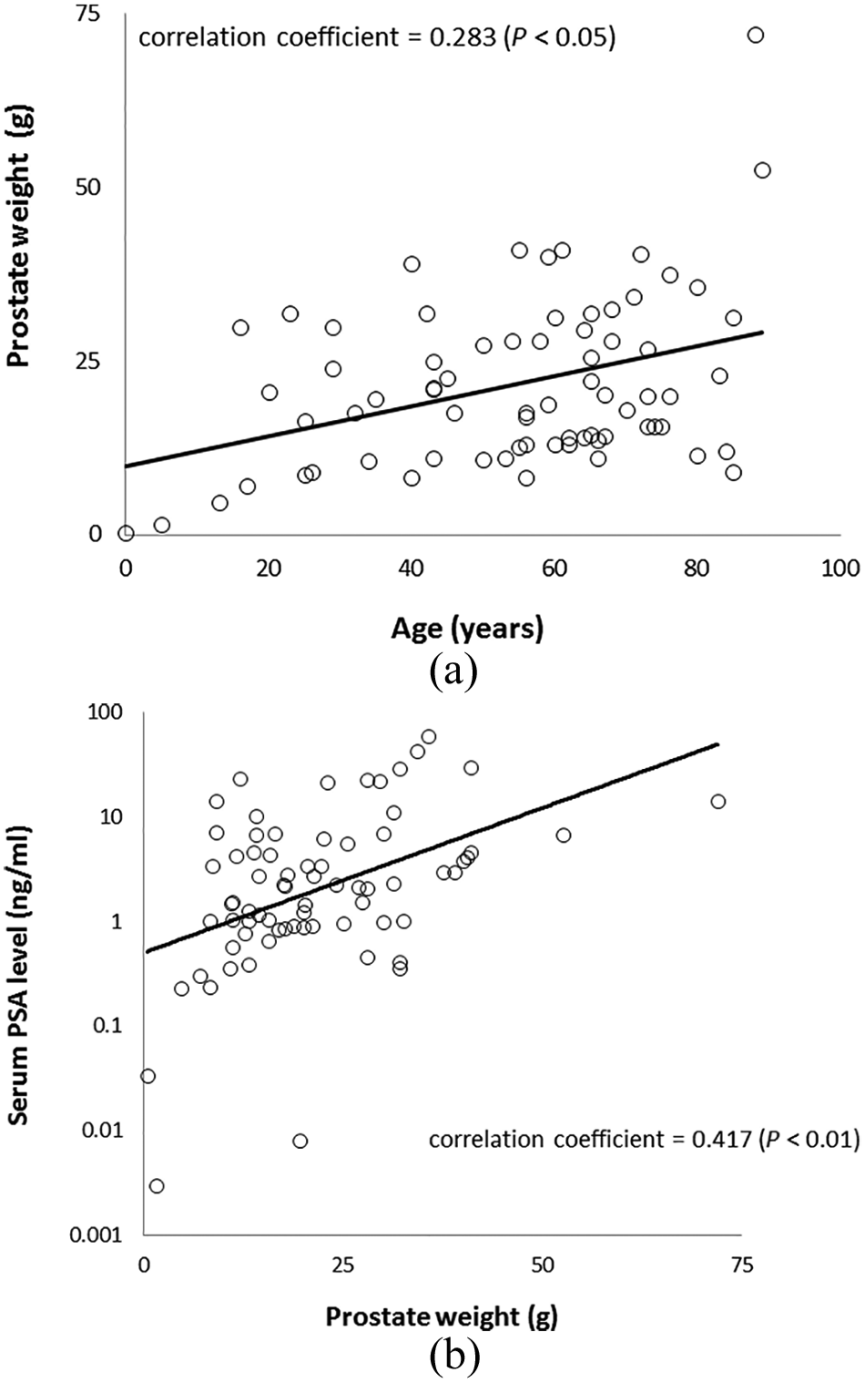
Correlation between prostate weight and age (n = 72). (a) Correlation between prostate weight and serum prostate-specific antigen (PSA) level (n = 72). (b) A significant weak positive correlation was observed in each case (Spearman’s rank correlation coefficient).

In the 72 cadavers, a significant positive correlation was observed between prostate weight and age (*P* < 0.05, correlation coefficient = 0.283) (Figure 3(a)). Similarly, a significant positive correlation was observed between prostate weight and postmortem serum PSA levels (*P* < 0.01, correlation coefficient = 0.417) (Figure 3(b)).

### Correlation between postmortem serum PSA levels and cause of death

In the 153 cadavers, no significant difference was observed between the cause of death and serum PSA levels among the seven groups classified by cause of death.

## Discussion

### Correlations between age and postmortem PSA levels

Previous studies have found that the morbidity of prostatic disease such as prostate cancer increases in proportion to age.^24^ In general, the serum PSA levels increase in prostatic disease. Accordingly, there is a possibility that the cause of high PSA levels in several cases was not due to aging, but rather due to the prostatic disease. However, in the present study, a significant positive correlation was observed between postmortem serum PSA levels and age after excluding cases of prostatic disease. Regarding the cause of an increase in serum PSA levels with age, Oesterling et al.^17^ suggested that this increase is caused by enlargement of prostate volume or an increase in permeability for PSA in ductal epithelium cells of the prostate. On the other hand, Yamazaki et al.^19^ showed a correlation between age and PSA density, suggesting that an enlargement of the prostate volume and the existence of prostatitis or infarction of the prostate leads to an increase in serum PSA levels with age. In the present study, the same weak positive correlations with age were observed in both serum PSA levels and prostate weight. Therefore, we hypothesized that an increase in prostate weight is primarily responsible for an increase in serum PSA levels with age.

### Relationship between PSA levels of cadavers and that of the living individuals

In the present study, several cadavers showed extremely high PSA levels, despite the subjects having no medical history of prostatic disease. It was thought that these high PSA levels were caused by deviation of PSA to blood associated with postmortem changes. However, no significant correlation was observed between postmortem interval and serum PSA levels. Jones et al.^25^ reported no statistically significant difference between serum PSA levels of antemortem and postmortem specimens of the same patient. Although antemortem PSA levels were not measured, not all postmortem cadavers showed abnormally high PSA levels in the present study. Thus, abnormally high PSA levels of cadavers are not caused by postmortem change, and it can be said that the postmortem PSA levels reflect the antemortem PSA levels. Therefore, we hypothesized that abnormally high PSA levels in cadavers are caused by several factors, such as prostate cancer, chronic or acute prostatitis, or stimulations of urethral catheter during resuscitation.

### Relationship between PMI and postmortem PSA levels

Forde et al.^20^ examined serum PSA levels after leaving specimens (blood collected from the same living patient) for various intervals (4, 8, 24, 48 h) at room temperature, and showed that there is no statistically significant difference in serum PSA levels among those four groups. While we could not precisely compare our results to that study because we used blood collected from cadavers, rather than living patients, our present findings that there was no significant correlation between serum PSA levels and PMI support their results. Therefore, PSA is considered as a stable material which is not influenced by putrefaction or by the environment of the cadavers.

### Relationship between BMI and postmortem PSA levels

Several previous reports have shown that BMI has a negative correlation with serum PSA levels.^26,27^ This correlation would influence the correlation between serum PSA levels and age, and could be a correction factor of age estimation of cadavers. On the other hand, other reports have shown that BMI has no correlation with serum PSA levels.^28–30^ Therefore, the opinion on this matter is divided. In the present study, no significant correlation was observed between serum PSA levels and BMI; therefore, we conclude that BMI has no influence on the correlation between serum PSA levels and age.

### Age at death estimation of cadavers based on serum PSA levels

Although the regression curve showed that the correlation between serum PSA levels and age (y = 0.1735e^0.0376x^, x: age, y: serum PSA levels) could allow us to estimate the age at death of unidentified cadavers, two cases in which this method should not be applied should be noted. The first case is that of infants. Serum PSA levels are often very low during childhood.^30,31^ Therefore, we should consider the possibility that the serum PSA levels during this period are unreliable for estimating age. The other case is that of the elderly. In the present study, the serum PSA levels generally increased with age, with two peaks in the twenties and sixties, reducing slightly in seventies, and the distribution of which varied in the elderly (Table 1). Although high PSA levels during the twenties might be caused by high sexual activity, the decline and variability of PSA levels in the elderly might be due to a different cause. Kirollos^32^ stated that

Although it is certain that serum PSA level correlates with age, the degree of the correlation is different among age groups. The correlation between PSA level and age are comparatively stronger in the fifties or sixties and becomes weaker after the seventies.

On the other hand, it is believed that the production of PSA is promoted due to the increase in prostate gland volume by the stimulation of androgen,^33^ and that the amount of androgen often decreases with age.^34^ Accordingly, it is thought that the number of cases with low serum PSA levels tend to increase with aging because of a decrease in the growing volume of prostate gland by decreased androgen. The level of androgen in elderlies may be depend on the sexual lifestyle in each individual. Thereby, in the elderly, it is thought that the distribution of PSA levels varies, and that the correlation between PSA levels and age tends to be weak.

According to our results and discussion above, the age estimation by serum PSA level might be more accurate in 20s–60s. Because of the low number of the autopsies in our area, our study size is very small. However, we showed a significant positive correlation between postmortem serum PSA levels and age of cadavers. The postmortem serum PSA levels are not dependent on the PMI, BMI, or the cause of death. Therefore, this correlation allows us to estimate the age of cadavers in an inexpensive, rapid, and objective manner. Accordingly, we revealed the possibility that measurement of serum PSA levels can be a very useful method for age estimation in the field of forensic medicine. However, because of the high RMSE value, the accuracy of age estimation of cadavers by serum PSA level is not so high. The precision of age estimation could be improved by combining this method with other methods such as using bones or teeth. In addition, the use of the method to measure PSA from whole blood that requires no centrifugation would allow us to examine PSA levels in hemolytic cases.^35^ Using such a method, it would be expected that there would be an increase in the number of cases in which age estimation of cadavers can be achieved using PSA levels. And our data may be also useful for research works of living individuals.

In the recent report, serum [−2]proPSA (p2PSA), a serum isoform of PSA is more accurate than the reference standard tests (tPSA, fPSA, and %fPSA) in predicting prostate cancer in men aged <60 years and may also be indicative of cancer aggressiveness.^36^ Therefore, we will try to analyze whether p2PSA is a better maker not only to detect Prostate cancer but also to estimate age.

## Conclusion

A significant positive correlation between postmortem serum PSA levels and age of cadavers.
